# Impact of Proton Pump Inhibitor Therapy on the Efficacy of Clopidogrel in the CAPRIE and CREDO Trials

**DOI:** 10.1161/JAHA.112.004564

**Published:** 2013-02-22

**Authors:** Steven P. Dunn, Steven R. Steinhubl, Deborah Bauer, Richard J. Charnigo, Peter B. Berger, Eric J. Topol

**Affiliations:** 1 University of Virginia Health System, Charlottesville, VA (S.P.D.); 2 Geisinger Clinic, Danville, PA (S.R.S., P.B.B.); 3 Sanofi, Bridgewater, NJ (D.B.); 4 University of Kentucky College of Public Health, Lexington, KY (R.J.C.); 5 Scripps Translational Science Institute, La Jolla, CA (E.J.T.)

**Keywords:** CAPRIE, clopidogrel, CREDO, drug–drug interaction, proton pump inhibitors

## Abstract

**Background:**

Proton pump inhibitors (PPIs) may interfere with the metabolic activation of clopidogrel via inhibition of cytochrome P450 2C19, but the clinical implications remain unclear.

**Methods and Results:**

The impact of PPI use on the 1‐year primary end point (ischemic stroke, myocardial infarction [MI], or vascular death) in the Clopidogrel versus Aspirin in Patients at Risk of Ischemic Events (CAPRIE) trial and the 28‐day (all‐cause death, MI, or urgent target vessel revascularization) and 1‐year (all‐cause death, MI, or stroke) primary end points in the Clopidogrel for Reduction of Events During Observation (CREDO) trial were examined. Clopidogrel appeared to elevate risk for the primary end point in CAPRIE among PPI users (estimated hazard ratio [EHR] 2.66, 95% CI 0.94 to 7.50) while lowering it for non‐PPI users (EHR 0.90, 95% CI 0.83 to 0.99, interaction *P*=0.047). Moreover, PPI use was associated with worse outcomes in patients receiving clopidogrel (EHR 2.39, 95% CI 1.74 to 3.28) but not aspirin (EHR 1.04, 95% CI 0.70 to 1.57, interaction *P*=0.001). Clopidogrel did not significantly alter risk for the 1‐year primary end point in CREDO among PPI users (EHR 0.82, 95% CI 0.48 to 1.40) while lowering it for non‐PPI users (EHR 0.71, 95% CI 0.52 to 0.98, interaction *P*=0.682). Also, PPI use was associated with worse outcomes in both patients receiving clopidogrel (EHR 1.67, 95% CI 1.06 to 2.64) and those receiving placebo (EHR 1.56, 95% CI 1.06 to 2.30, interaction *P*=0.811).

**Conclusions:**

In CREDO, the efficacy of clopidogrel was not significantly affected by PPI use. However, in CAPRIE, clopidogrel was beneficial to non‐PPI users while apparently harmful to PPI users. Whether this negative interaction is clinically important for patients receiving clopidogrel without aspirin needs further study.

## Introduction

Clopidogrel, a thienopyridine P2Y_12_ inhibitor of platelet function, is a cornerstone of cardiovascular pharmacotherapy, with aspirin, in the prevention of myocardial infarction (MI) and stent thrombosis after acute coronary syndromes and percutaneous coronary intervention (PCI). Clopidogrel is a prodrug, requiring conversion to an active metabolite that involves the cytochrome P450 (CYP) system via a combination of the isoenzymes CYP3A4, CYP1A2, CYP2C9, CYP2C19, and/or CYP2B6.^1^

The metabolic activation of clopidogrel via CYP450 has resulted in speculation about whether drugs that are inhibitors of or competitors for these isoenzymes may lessen the therapeutic effect of clopidogrel, especially via CYP2C19. Many studies have suggested that such drugs have a significant impact on clopidogrel's inhibition of ex vivo measurements of platelet function,^2–3^ but the clinical impact of this has been difficult to demonstrate.^4–5^ Such an impact would be important to identify with proton pump inhibitors (PPIs), many of which inhibit CYP2C19, because PPIs are recommended as first‐line therapy to prevent gastrointestinal complications in high‐risk patients.^6^ Accordingly, both clopidogrel and PPIs are among the most highly prescribed drugs in the world, and they are often coadministered.^7^ The potential for this interaction was first reported by Gilard and colleagues,^2^ where patients undergoing PCI using clopidogrel who were receiving PPIs were found to have higher levels of platelet reactivity than patients not receiving PPIs. However, clinical data regarding this interaction have been variable, with some,^8–10^ but not all,^11–13^ observational studies of databases or claims registries demonstrating an increase in cardiovascular events with coadministration, whereas analyses of clinical trials have been more uniform in demonstrating no significant clinical interaction.^5,14^ The significance of this interaction continues to be debated, with both the US Food and Drug Administration and the European Medicines Agency recommending that clopidogrel and CYP2C19 inhibitors should not be administered with PPIs, specifically indicating esomeprazole and omeprazole.^15–16^

Accordingly, we sought to examine whether PPIs affected clopidogrel efficacy in the only 2 major placebo‐controlled clinical trials of clopidogrel as an active treatment strategy in which PPI use was documented—in the Clopidogrel for Reduction of Events During Observation (CREDO) study and the Clopidogrel versus Aspirin in Patients at Risk of Ischemic Events (CAPRIE) study.

## Methods

This study was a post hoc, retrospective analysis of the CREDO and CAPRIE trials, the details of which have been previously published.^17–18^ Briefly, the CREDO trial was a prospective, randomized, double‐blind, placebo‐controlled trial of 2116 patients at high likelihood of undergoing PCI that was conduct between 1999 and 2001. Patients were randomized between 3 and 24 hours before PCI to receive either a 300‐mg loading dose of clopidogrel or placebo. Afterward, all patients received open label clopidogrel for 28 days. After 28 days, patients randomized to the clopidogrel loading dose received clopidogrel 75 mg/d for the next 11 months; patients initially randomized to placebo loading dose received placebo daily for 11 months. The coprimary end points of the trial were the 28‐day composite incidence of all‐cause death, MI, or urgent target vessel revascularization and the 1‐year composite incidence of all‐cause death, MI, or stroke. The CAPRIE trial was a randomized, prospective, controlled trial of 19 185 high‐risk patients with either prior MI, ischemic stroke, or peripheral vascular disease, who were randomized between 1992 and 1995 to receive monotherapy with either aspirin 325 mg daily or clopidogrel 75 mg daily. The primary outcome was the composite end point of ischemic stroke, MI, or vascular death after a minimum of 1 year of treatment.

In addition, we performed a meta‐analysis in which these new data were combined with the available published data sets from blinded, randomized, controlled trials comparing clopidogrel with a treatment believed not to be influenced by concomitant PPI use in which PPI use data were available. These trials include CAPRIE, CREDO, the TRial to assess Improvement in Therapeutic Outcomes by optimizing platelet iNhibition with prasugrel (TRITON) trial, and the PLATelet inhibition and patient Outcomes (PLATO) trial. The goal of the meta‐analysis was to obtain overall point and interval estimates for adjusted hazard ratios comparing PPI users with non–PPI users on the trials' primary end points, among patients randomized to clopidogrel and not randomized to clopidogrel. The TRITON and PLATO trials had a primary end point of cardiovascular death, MI, or stroke, and patients not randomized to clopidogrel in these trials were randomized to prasugrel and ticagrelor, respectively.

The authors had full access to the data for the primary analysis with the CAPRIE and CREDO trials and take responsibility for its integrity. All authors have read and agree to the article as it is written.

### PPI Use

The decision to treat with a PPI in either trial was made at the discretion of the patient's provider and was not required by protocol. PPI use was identified both at study baseline and at each study follow‐up, along with other concomitant medication use. For the purposes of examining the impact of PPI use on outcomes in CAPRIE and CREDO, patients were defined as PPI users if they were receiving a PPI (omeprazole, lansoprazole, rabeprazole, pantoprazole, or esomeprazole) as active treatment. Active PPI treatment was examined as a time‐dependent covariate, where if PPI use was documented at a study visit, the start date was assumed to be 1 day after the previous “No” visit. Subsequently, if PPI use was later found to be discontinued at a visit, then the stop date was assumed to be 1 day after the previous “Yes” visit. This analysis was performed because PPI use was potentially of limited duration in adherence with the original omeprazole product label recommendation of short‐term treatment courses for reflux disease and other gastrointestinal disorders.^19^ This potential limitation was thought to primarily affect the CAPRIE analysis, which had a significantly lower percentage of PPI users than the CREDO population and was conducted several years earlier. Additional sensitivity analyses were performed varying the definition of PPI use, including defining PPI use at study baseline, to preserve the randomized comparison, and PPI use at any point throughout study follow‐up. Moreover, besides comparing PPI use with non–PPI use within treatment strata, we also directly compared clopidogrel use with non–clopidogrel use within PPI strata.

### Statistical Analysis

Numerical baseline characteristics are presented as mean and SD values and were stratified based on PPI use at study baseline. Categorical baseline characteristics, similarly stratified, are summarized as numbers and percentages. Comparison tests for each baseline characteristic were performed with either the χ^2^ test for categorical variables or ANOVA (type 3) for continuous variables. Records with missing values on variables examined herein were excluded from analysis.

In CAPRIE and CREDO, the primary end points were evaluated comparing clopidogrel use with non–clopidogrel use within PPI strata using Cox proportional hazards models. Patients in CAPRIE were additionally stratified by qualifying condition. Additional analyses were conducted using a log‐rank test comparing patients receiving a PPI, defined as active PPI treatment, with those patients not taking a PPI in each treatment group. The unadjusted hazard ratio comparing PPI users with non–PPI users and 95% CIs were estimated within treatment strata using a Cox proportional hazards model. To reduce selection bias, we also estimated adjusted hazard ratios comparing PPI users with non–PPI users within treatment strata. For this purpose, the propensity to receiving a PPI at baseline was first estimated via multivariable logistic regression. In CAPRIE, the propensity scores were calculated conditional on race, diabetes, hypercholesterolemia, congestive heart failure, cardiomegaly, atrial fibrillation, stable angina, unstable angina, previous MI, transient ischemic attack, reversible ischemic neurological deficit, previous ischemic stroke, intermittent claudication, and leg amputation. In CREDO, the propensity scores were calculated conditional on race, diabetes, hyperlipidemia, congestive heart failure, atrial fibrillation, stable angina, unstable angina, previous MI, previous ischemic stroke, peripheral vascular disease, PCI, coronary angiography, and coronary artery bypass grafting. The adjustment characteristics in CAPRIE were chosen due to their previous association with ischemic outcomes in the CAPRIE trial.^20^ Comparable covariates and significant covariates (*P*<0.1) between baseline PPI‐ versus non–PPI‐treated patients were used to determine adjustment variables in CREDO. Each Cox proportional hazards model was adjusted for covariates described earlier and stratified by 5 propensity score strata; in CAPRIE, the qualifying condition for enrollment was also a stratification factor. The CAPRIE and CREDO 1‐year analyses were intention‐to‐treat analyses, whereas the CREDO 28‐day analysis was per protocol and included all patients who received a loading dose and who underwent PCI.

In adddition, we performed a random‐effects meta‐analysis using the DerSimonian–Laird method to obtain overall point estimates and 95% CIs for adjusted hazard ratios comparing PPI users with non–PPI users,^20^ both among patients randomized to clopidogrel and among patients not randomized to clopidogrel, using data from the CAPRIE, CREDO, TRITON, and PLATO trials. Point estimates and 95% CIs for adjusted hazard ratios in the TRITON and PLATO trials were extracted from published articles examining PPI use in these trials.^5,21^

## Results

### CAPRIE

#### Baseline characteristics

Of the 19 185 patients in CAPRIE, 218 (1.1%) were receiving a PPI at study entry. Of these, 216 patients were receiving omeprazole and 2 patients were receiving lansoprazole, both strong CYP2C19 inhibiting PPIs. Table 1 depicts the baseline characteristics collected in CAPRIE stratified by baseline PPI use. Overall, patients receiving a PPI at study baseline were more likely to have a body mass index >25 kg/m^2^, stable angina, and were more likely to be enrolled in the CAPRIE trial on the basis of a prior MI.

**Table 1.** CAPRIE and CREDO Demographic and Other Baseline Data by Baseline PPI Use

CAPRIE	No PPI (n=18 967)	PPI (n=218)	*P* Value
Age, mean (SD), y	62.5 (11.1)	63.9 (11.0)	0.056
Women, No. (%)	5263 (27.7)	67 (30.7)	0.328
Race, No. (%)			
White	17 968 (94.7)	209 (95.9)	0.648
Black	548 (2.9)	4 (1.8)	
Other	451 (2.4)	5 (2.3)	
Weight, mean (SD), kg	76.6 (14.7)	77.7 (14.2)	0.270
Body mass index, mean kg/m^2^ (SD)	26.5 (4.4)	26.7 (4.1)	0.372
>25 kg/m^2^, No. (%)	11 516 (60.8)	147 (67.4)	0.047
Risk factors and cardiovascular history, No. (%)			
Smoking history, current	5616 (29.6)	52 (23.9)	0.064
Hypertension	9777 (51.5)	108 (49.5)	0.556
Diabetes	3845 (20.3)	36 (16.5)	0.170
Hypercholesterolemia	7818 (41.2)	86 (39.4)	0.600
Stable angina	4101 (21.6)	71 (32.6)	<0.001
Unstable angina	1635 (8.6)	26 (11.9)	0.084
Atrial fibrillation	803 (4.2)	8 (3.7)	0.681
Cardiac surgery	1469 (7.7)	20 (9.2)	0.433
Other cardiac arrhythmia	2017 (10.6)	26 (11.9)	0.539
Cardiac valve disease	736 (3.9)	11 (5.0)	0.376
Cardiomegaly	876 (4.6)	9 (4.1)	0.732
Heart failure	1061 (5.6)	13 (6.0)	0.814
Qualifying condition, No. (%)			
Ischemic stroke	6373 (33.6)	58 (26.6)	0.048
Peripheral artery disease	6378 (33.6)	74 (33.9)	
MI	6216 (32.8)	86 (39.4)	

CREDO	No PPI (n=1742)	PPI (n=374)	
Age, mean (SD), y	61.6 (11.1)	61.8 (11.0)	0.707
Woman, No. (%)	495 (28.4)	111 (29.7)	0.624
Race, No. (%)			
White	1546 (88.7)	334 (89.3)	0.388
Black	100 (5.7)	23 (6.1)	
Hispanic	83 (4.8)	12 (3.2)	
Other	13 (0.7)	5 (1.3)	
Risk factors and cardiovascular history, No. (%)			
Smoking within past year	539 (30.9)	108 (28.9)	0.432
Diabetes	458 (26.3)	102 (27.3)	0.696
Family history of premature CAD	730 (41.9)	162 (43.3)	0.616
Hyperlipidemia	1279 (73.4)	296 (79.1)	0.021
CABG	266 (15.3)	69 (18.4)	0.126
Coronary angiography	1275 (73.2)	304 (81.3)	0.001
PCI	458 (26.3)	132 (35.3)	<0.001
Atrial fibrillation	78 (4.5)	18 (4.8)	0.777
Cerebrovascular disease	104 (6.0)	22 (5.9)	0.948
Congestive heart failure	152 (8.7)	33 (8.8)	0.952
Hypertension	1192 (68.4)	258 (69.0)	0.833
MI	579 (33.2)	139 (37.2)	0.146
Peripheral vascular disease	135 (7.7)	41 (11.0)	0.041
Previous stroke	118 (6.8)	23 (6.1)	0.661
Indication for PCI, No. (%)			
Recent MI	231 (13.3)	59 (15.8)	0.199
Stable angina and other	602 (34.6)	92 (24.6)	<0.001
Unstable angina	896 (51.4)	221 (59.1)	0.007

CAPRIE indicates Clopidogrel versus Aspirin in Patients at Risk of Ischemic Events; CREDO, Clopidogrel for Reduction of Events During Observation; PPI, proton pump inhibitor; MI, myocardial infarction; CAD, coronary artery disease; CABG, coronary artery bypass grafting; PCI, percutaneous coronary intervention.

#### Primary end point

Among patients receiving a PPI at study baseline (n=218), the rate of the primary end point (ischemic stroke, MI, or vascular death) was 11.7% in patients randomly assigned to clopidogrel compared with 4.7% in patients randomly assigned to receive aspirin (unadjusted EHR 2.66, 95% CI 0.94 to 7.50) (Table 2). In patients not receiving a PPI at study baseline (n=18 967), the rate of the primary end point was 9.8% in patients receiving clopidogrel compared with 10.7% in patients receiving aspirin (unadjusted EHR 0.90, 95% CI 0.83 to 0.99); thus, there was a significant interaction (*P*=0.047, Table 2).

**Table 2.** CAPRIE—Primary Efficacy Analysis Stratified by PPI Type Using Various Definitions for PPI Treatment

	Subgroup	Clopidogrel, n (%)	Aspirin, n (%)	Unadjusted Estimated HR (95% CI)	*P* Value for Interaction
Baseline PPI use					
Any PPI	No (n=18 967)	926 (9.8)	1015 (10.7)	0.90 (0.83 to 0.99)	0.047
	Yes (n=218)	13 (11.7)	5 (4.7)	2.66 (0.94 to 7.50)	
Omeprazole	No (n=18 969)	926 (9.8)	1016 (10.7)	0.90 (0.83 to 0.99)	0.027
	Yes (n=216)	13 (11.8)	4 (3.8)	3.37 (1.09 to 10.4)	
Lansoprazole	No (n=19 183)	939 (9.8)	1019 (10.6)	0.91 (0.84 to 1.00)	N/A
	Yes (n=2)	0	1 (100)	N/A	
Concomitant PPI use					
Any PPI	No (n=18 316)	884 (9.6)	975 (10.7)	0.89 (0.81 to 0.98)	0.019
	Yes (n=869)	55 (13.8)	45 (9.6)	1.41 (0.95 to 2.09)	
Omeprazole	No (n=18 340)	884 (9.6)	976 (10.7)	0.89 (0.81 to 0.98)	0.015
	Yes (n=845)	55 (14.1)	44 (9.6)	1.44 (0.96 to 2.14)	
Lansoprazole	No (n=19 148)	938 (9.8)	1018 (10.6)	0.91 (0.84 to 1.00)	0.774
	Yes (n=37)	1 (6.7)	2 (9.1)	0.58 (0.05 to 6.43)	
Any PPI use					
Any PPI	No (n=18 298)	882 (9.6)	975 (10.7)	0.89 (0.81 to 0.97)	0.011
	Yes (n=887)	57 (14.0)	45 (9.4)	1.46 (0.99 to 2.16)	
Omeprazole	No (n=18 322)	882 (9.6)	976 (10.7)	0.89 (0.81 to 0.97)	0.009
	Yes (n=863)	57 (14.3)	44 (9.5)	1.49 (1.00 to 2.21)	
Lansoprazole	No (n=19 148)	938 (9.8)	1018 (10.6)	0.91 (0.84 to 1.00)	0.774
	Yes (n=37)	1 (6.7)	2 (9.1)	0.58 (0.05 to 6.43)	

CAPRIE indicates Clopidogrel versus Aspirin in Patients at Risk of Ischemic Events; PPI, proton pump inhibitor; HR, hazard ratio; N/A, not available.

In patients randomly assigned to receive clopidogrel (n=9599), the rate of the primary end point was 14.0% in patients receiving a PPI as active treatment compared with 9.6% in patients not receiving a PPI (unadjusted EHR based on time‐varying covariate 2.66, 95% CI 1.94 to 3.63, *P*<0.001) (Table 3). For patients randomly assigned to aspirin (n=9586), the rate of the primary end point in patients receiving a PPI was 9.4% versus 10.7% in patients not receiving a PPI (unadjusted EHR 1.17, 95% CI 0.78 to 1.76, *P*=0.439) (Table 3). After adjustment for covariates listed in the caption to Table 3 and stratification by propensity scores, PPI use remained significantly associated with the primary end point in patients receiving clopidogrel (adjusted EHR based on time‐varying covariate 2.39, 95% CI 1.74 to 3.28, *P*<0.001) but not in those receiving aspirin (adjusted EHR 1.04, 95% CI 0.70 to 1.57, *P*=0.834; *P* for interaction=0.001).

**Table 3.** CAPRIE—Unadjusted and Adjusted* EHRs (95% CI) for the Primary Efficacy End Point by PPI Use (Time‐Dependent Variable)

	Clopidogrel				Aspirin				*P* Value for Interaction*
	PPI	No PPI	Unadjusted EHR (95% CI)	Adjusted EHR (95% CI)	PPI	No PPI	Unadjusted EHR (95% CI)	Adjusted EHR (95% CI)	
IS, MI, vascular death	14.0% (57/408)	9.6% (882/9191)	2.66 (1.94 to 3.63), *P*<0.001	2.39 (1.74 to 3.28), *P*<0.001	9.4% (45/479)	10.7% (975/9107)	1.17 (0.78 to 1.76), *P*=0.439	1.04 (0.70 to 1.57), *P*=0.834	0.001

CAPRIE indicates Clopidogrel versus Aspirin in Patients at Risk of Ischemic Events; EHR, estimated hazard ratio; PPI, proton pump inhibitor; MI, myocardial infarction; RIND, reversible ischemic neurological deficit; IS, ischemic stroke.

* Adjusted model includes race, diabetes, hypercholesterolemia, congestive heart failure, cardiomegaly, atrial fibrillation, stable angina, unstable angina, previous MI, TIA, RIND, previous IS, intermittent claudication, and leg amputation. Both models are stratified by qualifying condition, and the adjusted model is additionally stratified by 5 propensity score strata.

* Interaction analysis performed on adjusted comparison.

Additional analyses were conducted, varying the definition of PPI use. Among patients not taking PPI at any time during the study, those randomized to clopidogrel had an estimated 11% lesser hazard on the primary end point than those randomized to aspirin; in contrast, among patients taking PPIs at any time during the study, those randomized to clopidogrel had an estimated 46% greater hazard on the primary end point than those randomized to aspirin, yielding a statistically significant interaction (*P*=0.011, Table 2). After adjustment for confounders and stratifying by propensity scores, any PPI use was significantly associated with the primary end point in patients receiving clopidogrel (adjusted EHR 1.34, 95% CI 1.02 to 1.76, *P*=0.033) but not aspirin (adjusted EHR 0.81, 95% CI 0.60 to 1.09, *P*=0.157).

### CREDO

#### Baseline characteristics

Of the 2116 patients enrolled in CREDO, 374 (17.7%) patients were receiving a PPI at study entry, a higher percentage of patients compared with the CAPRIE population (1.1%). Of these, the majority of patients were receiving lansoprazole (n=218), followed by omeprazole (n=155), pantoprazole (n=15), and rabeprazole (n‐9); 23 patients were receiving ≥2 PPIs at study entry. Table 1 depicts the baseline characteristics stratified by PPI use at study baseline. Of note, patients receiving a PPI were more likely to have a history of hyperlipidemia, PCI, coronary angiography, and/or peripheral vascular disease. In addition, PPI users were more likely to have recently had unstable angina than were those not taking a PPI at study entry.

#### Primary end point

Among patients who were receiving a PPI at study baseline (n=336), the rate of the 28‐day primary end point (all‐cause death, MI, urgent target vessel revascularization) in patients receiving a clopidogrel loading dose was 11.1% compared with 10.3% in patients receiving a placebo loading dose (unadjusted EHR 1.10, 95% CI 0.57 to 2.11) (Table 4). In patients who were not receiving a PPI at study baseline (n=1479), the rate of the 28‐day primary end point was 5.8% in patients receiving a clopidogrel loading dose compared with 7.8% in patients receiving a placebo loading dose (unadjusted EHR 0.74, 95% CI 0.50 to 1.10); thus, there was no significant interaction (*P*=0.315, Table 4).

**Table 4.** CREDO—Primary Efficacy Analysis (28‐Day) Stratified by PPI Type Using Various Definitions for PPI Treatment

	Subgroup	Clopidogrel LD, n (%)	Placebo LD, n (%)	Estimated Unadjusted HR (95% CI)	*P* Value for Interaction
Baseline PPI use					
Any PPI	No (n=1479)	43 (5.8)	58 (7.8)	0.74 (0.50 to 1.10)	0.315
	Yes (n=336)	18 (11.1)	18 (10.3)	1.10 (0.57 to 2.11)	
Omeprazole	No (n=1676)	54 (6.5)	70 (8.3)	0.78 (0.55 to 1.12)	0.464
	Yes (n=139)	7 (10.1)	6 (8.6)	1.20 (0.40 to 3.58)	
Lansoprazole	No (n=1618)	49 (6.1)	63 (7.8)	0.78 (0.54 to 1.13)	0.524
	Yes (n=197)	12 (12.8)	13 (12.6)	1.03 (0.47 to 2.27)	
Pantoprazole	No (n=1800)	61 (6.8)	75 (8.3)	0.83 (0.59 to 1.16)	N/A
	Yes (n=15)	0 (0.00)	1 (14.3)	N/A	
Rabeprazole	No (n=1810)	61 (6.8)	76 (8.3)	0.81 (0.58 to 1.14)	N/A
	Yes (n=5)	0	0	N/A	
Any PPI use					
Any PPI	No (n=1262)	34 (5.4)	50 (7.9)	0.67 (0.43 to 1.04)	0.141
	Yes (n=553)	27 (10.1)	26 (9.1)	1.13 (0.66 to 1.94)	
Omeprazole	No (n=1561)	50 (6.5)	66 (8.3)	0.78 (0.54 to 1.13)	0.586
	Yes (n=254)	11 (8.3)	10 (8.3)	1.02 (0.43 to 2.39)	
Lansoprazole	No (n=1486)	41 (5.5)	59 (7.9)	0.70 (0.47 to 1.04)	0.121
	Yes (n=329)	20 (12.4)	17 (10.1)	1.27 (0.66 to 2.42)	
Pantoprazole	No (n=1760)	58 (6.6)	71 (8.0)	0.83 (0.59 to 1.17)	0.688
	Yes (n=55)	3 (11.1)	5 (17.9)	0.62 (0.15 to 2.61)	
Rabeprazole	No (n=1786)	60 (6.7)	76 (8.5)	0.79 (0.57 to 1.11)	N/A
	Yes (n=29)	1 (10.0)	0	N/A	

CREDO indicates Clopidogrel for Reduction of Events During Observation; PPI, proton pump inhibitor; LD, loading dose; HR, hazard ratio; N/A, not available.

In patients receiving a clopidogrel loading dose (n=900), the rate of the 28‐day primary end point in patients receiving a PPI as active treatment was 10.1% compared with 5.4% in patients not receiving a PPI (unadjusted EHR based on time‐varying covariate 1.82, 95% CI 1.10 to 3.04, *P*=0.021) (Table 5). In contrast, among patients receiving a placebo loading dose (n=915), the rate of the primary end point in those receiving a PPI as active treatment was 9.1% compared with 7.9% in patients not receiving a PPI (unadjusted EHR 1.16, 95% CI 0.72 to 1.88, *P*=0.538) (Table 5). After adjustment for covariates listed in the caption to Table 5 and stratification by propensity scores, PPI use remained significantly associated with the primary end point in patients receiving a clopidogrel loading dose (adjusted EHR based on time‐varying covariate 1.71, 95% CI 1.09 to 2.91, *P*=0.047) but not a placebo loading dose (adjusted EHR 1.13, 95% CI 0.70 to 1.84, *P*=0.617; *P* for interaction=0.258).

**Table 5.** CREDO—28‐Day Primary End Point Unadjusted and Adjusted* Estimated Hazard Ratios (95% CI) by PPI Use (Time‐Dependent Variable)

	Clopidogrel+ASA (Clopidogrel LD)				Clopidogrel+ASA (Placebo LD)				*P* Value for Interaction*
	PPI	No PPI	Unadjusted EHR (95% CI)	Adjusted EHR (95% CI)	PPI	No PPI	Unadjusted EHR (95% CI)	Adjusted EHR (95% CI)	
All‐cause death, MI, urgent target vessel revascularization	10.1% (27/268)	5.4% (34/632)	1.82 (1.10 to 3.04), *P*=0.021	1.71 (1.09 to 2.91), *P*=0.047	9.1% (26/285)	7.9% (50/630)	1.16 (0.72 to 1.88), *P*=0.538	1.13 (0.70 to 1.84), *P*=0.617	0.258

CREDO indicates Clopidogrel for Reduction of Events During Observation; PPI, proton pump inhibitor; ASA, aspirin; LD, loading dose; EHR, estimated hazard ratio; MI, myocardial infarction; PCI, percutaneous coronary intervention; IS, ischemic stroke; CABG, coronary artery bypass grafting.

* Adjusted model includes race, diabetes, hyperlipidemia, congestive heart failure, atrial fibrillation, stable angina, unstable angina, previous MI, previous IS, peripheral vascular disease, PCI, coronary angiography, and CABG and is stratified by 5 propensity score strata.

* Interaction analysis performed on adjusted comparison.

Among patients not taking PPIs at any time during the study, those randomized to clopidogrel had an estimated 33% lesser hazard on the primary end point than those randomized to placebo; among patients taking PPI at any time during the study, those randomized to clopidogrel had an estimated 13% greater hazard on the primary end point than those randomized to placebo, so that the interaction was not statistically significant (*P*=0.141, Table 4). After adjustment for potential confounders and stratifying by propensity scores, any PPI use was associated with the 28‐day primary end point in patients randomized to the clopidogrel loading dose (adjusted EHR 1.81, 95% CI 1.07 to 3.05, *P*=0.026) but not the placebo loading dose (adjusted EHR 1.10, 95% CI 0.68 to 1.79, *P*=0.692).

For the 1‐year primary end point, in patients receiving a PPI at baseline (n=374), the rate of the primary end point (all‐cause death, MI, stroke) in patients randomized to clopidogrel was 12.8% compared with 15.9% in patients randomized to placebo (unadjusted EHR 0.82, 95% CI 0.48 to 1.40) (Table 6). In patients not receiving a PPI at baseline (n=1742), the rate of the primary end point in patients randomized to clopidogrel was 7.6% compared with 10.5% in patients randomized to placebo (unadjusted EHR 0.71, 95% CI 0.52 to 0.98); thus, there was no significant interaction (*P*=0.682, Table 6).

**Table 6.** CREDO—Primary Efficacy Analysis (1 Year) Stratified by PPI Type Using Various Definitions for PPI Treatment

	Subgroup	Clopidogrel, n (%)	Placebo, n (%)	Estimated Unadjusted HR (95% CI)	*P* Value for Interaction
Baseline PPI use					
Any PPI	No (n=1742)	66 (7.6)	91 (10.5)	0.71 (0.52 to 0.98)	0.682
	Yes (n=374)	23 (12.8)	31 (15.9)	0.82 (0.48 to 1.40)	
Omeprazole	No (n=1961)	80 (8.2)	112 (11.4)	0.71 (0.54 to 0.95)	0.602
	Yes (n=155)	9 (11.7)	10 (12.8)	0.93 (0.38 to 2.28)	
Lansoprazole	No (n=1898)	74 (7.8)	102 (10.7)	0.72 (0.53 to 0.97)	0.721
	Yes (n=218)	15 (14.4)	20 (17.5)	0.82 (0.42 to 1.61)	
Pantoprazole	No (n=2101)	88 (8.4)	118 (11.2)	0.75 (0.57 to 0.99)	0.202
	Yes (n=15)	1 (12.5)	4 (57.1)	0.13 (0.01 to 1.18)	
Rabeprazole	No (n=2107)	89 (8.5)	122 (11.5)	0.73 (0.55 to 0.96)	N/A
	Yes (n=9)	0	0	N/A	
Any PPI use					
Any PPI	No (n=1490)	53 (7.0)	74 (10.0)	0.69 (0.49 to 0.99)	0.551
	Yes (n=626)	36 (12.0)	48 (14.8)	0.82 (0.53 to 1.26)	
Omeprazole	No (n=1826)	72 (8.0)	103 (11.2)	0.71 (0.52 to 0.95)	0.578
	Yes (n=290)	17 (11.5)	19 (13.4)	0.86 (0.45 to 1.66)	
Lansoprazole	No (n=1754)	63 (7.2)	91 (10.4)	0.68 (0.49 to 0.94)	0.348
	Yes (n=362)	26 (14.9)	31 (16.6)	0.91 (0.54 to 1.54)	
Pantoprazole	No (n=2054)	85 (8.3)	113 (10.9)	0.76 (0.57 to 1.00)	0.271
	Yes (n=62)	4 (12.5)	9 (30.0)	0.38 (0.12 to 1.25)	
Rabeprazole	No (n=2081)	89 (8.6)	122 (11.7)	0.72 (0.55 to 0.95)	N/A
	Yes (n=35)	0	0	N/A	

CREDO indicates Clopidogrel for Reduction of Events During Observation; PPI, proton pump inhibitor; HR, hazard ratio; N/A, not available.

In patients randomized to clopidogrel (n=1053), the rate of the primary end point in patients receiving a PPI as active treatment was 12.0% compared with 7.0% in patients not receiving a PPI (unadjusted EHR based on time‐varying covariate 1.68, 95% CI 1.07 to 2.63, *P*=0.024) (Table 7). Among patients randomized to placebo (n=1063), the rate of the primary end point in those receiving a PPI was 14.8% compared with 10.0% in patients not receiving a PPI (unadjusted EHR 1.61, 95% CI 1.10 to 2.35, *P*=0.014) (Table 7). After adjustment for covariates listed in the caption to Table 7 and stratification by propensity scores, PPI use was still significantly associated with the primary end point in patients receiving both clopidogrel (adjusted EHR based on time‐varying covariate 1.67, 95% CI 1.06 to 2.64, *P*=0.027) and placebo (adjusted EHR 1.56, 95% CI 1.06 to 2.30, *P*=0.025; *P* for interaction=0.811).

**Table 7.** CREDO—1‐Year Primary End Point Unadjusted and Adjusted* Estimated Hazard Ratios (95% CI) by PPI Use (Time‐Dependent Variable)

	Clopidogrel+ASA				Placebo+ASA				*P* Value for Interaction*
	PPI	No PPI	Unadjusted EHR (95% CI)	Adjusted EHR (95% CI)	PPI	No PPI	Unadjusted EHR (95% CI)	Adjusted EHR (95% CI)	
All‐cause death, MI, stroke	12.0% (36/301)	7.0% (53/752)	1.68 (1.07 to 2.63), *P*=0.024	1.67 (1.06 to 2.64), *P*=0.027	14.8% (48/325)	10.0% (74/738)	1.61 (1.10 to 2.35), *P*=0.014	1.56 (1.06 to 2.30), *P*=0.025	0.811

CREDO indicates Clopidogrel for Reduction of Events During Observation; PPI, proton pump inhibitor; ASA, aspirin; EHR, estimated hazard ratio; MI, myocardial infarction; PCI, percutaneous coronary intervention; IS, ischemic stroke; CABG, coronary artery bypass grafting.

* Adjusted model includes race, diabetes, hyperlipidemia, congestive heart failure, atrial fibrillation, stable angina, unstable angina, previous MI, previous IS, peripheral vascular disease, PCI, coronary angiography, and CABG and is stratified by 5 propensity score strata.

* Interaction analysis performed on adjusted comparison.

Among patients not taking PPIs at any time during the study, those randomized to clopidogrel had an estimated 31% lesser hazard for reaching the primary end point than those randomized to placebo; among patients taking PPIs at any time during the study, those randomized to clopidogrel had an estimated 18% lesser hazard of reaching the primary end point than those randomized to placebo, so that the interaction was not statistically significant (*P*=0.551, Table 6). After adjustment for potential confounders and stratifying by propensity scores, any PPI use was associated with the primary event in both patients randomized to clopidogrel (adjusted EHR 1.76, 95% CI 1.14 to 2.71, *P*=0.010) and to placebo (adjusted EHR 1.49, 95% CI 1.03 to 2.16, *P*=0.035).

### Meta‐analysis

**Figure 1.**
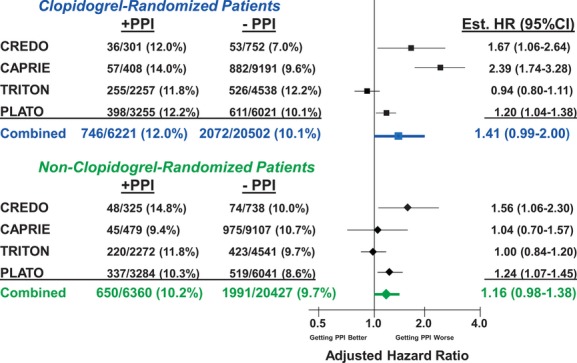
Meta‐analysis of the effect of PPI use on primary outcomes in randomized controlled trials of clopidogrel vs non–clopidogrel antiplatelet therapy**.** Data from the 53 510 patients enrolled in the 4 randomized controlled trials of clopidogrel (CAPRIE, CREDO, TRITON, PLATO) were included in the meta‐analysis. The vertical line indicates an adjusted hazard ratio (HR) of 1.0. EHR comparing PPI users with non–PPI users for the trial's primary end point for patients randomized to clopidogrel (■) or for patients randomized to non–clopidogrel antiplatelet therapy (♦). The horizontal lines indicate corresponding 95% CIs. Meta‐analysis estimates are presented in bold. The overall *P* value for interaction between PPI use and clopidogrel was 0.39. PPI indicates proton pump inhibitor; CAPRIE, Clopidogrel versus Aspirin in Patients at Risk of Ischemic Events; CREDO, Clopidogrel for Reduction of Events During Observation; TRITON, TRial to assess Improvement in Therapeutic Outcomes by optimizing platelet iNhibition with prasugrel; PLATO, PLATelet inhibition and patient Outcomes; EHR, estimated hazard ratio.

Data from the 53 510 patients enrolled in the 4 randomized controlled trials of clopidogrel were included in the meta‐analysis, including 26 723 randomized to daily clopidogrel and 26 787 randomized to the alternative antiplatelet agent whose metabolism is not believed to be influenced by PPI use. In total, 12 581 of enrolled patients received a PPI (23.5%), whereas 40 929 did not. Among patients randomized to clopidogrel, the overall estimate (and 95% CI) for an adjusted hazard ratio comparing PPI users with non–PPI users was 1.41 (0.99 to 2.00; *P*=0.053) (Figure 1). Among patients randomized to the alternative antiplatelet agent, the overall estimate (and 95% CI) was 1.16 (0.98 to 1.38; *P*=0.08) (Figure 1). The overall *P* value for interaction between PPI use and clopidogrel was 0.39. These findings can be understood by noting that (1) CREDO and PLATO both found a positive association between PPI use and occurrence of the primary end point, regardless of assigned antiplatelet agent; (2) TRITON found essentially no association between PPI use and the primary end point of the trial in either arm; and (3) CAPRIE found a positive association between PPI use and the primary end point among patients randomized to clopidogrel but essentially no association between PPI use and the primary end point among patients randomized to aspirin.

## Discussion

The most important finding of this study was that the use of PPIs was associated with adverse outcomes in patients randomized to clopidogrel in the CAPRIE trial but not in those randomized to aspirin. In contrast, PPI use was associated with adverse outcomes in both clopidogrel and placebo groups in patients enrolled in the CREDO trial.

The data from the CREDO trial are consistent with both observational and clinical trial data that have been previously published. Some,^8–10^ but not all,^11–13^ observational analyses of claims databases or registries indicate an association of adverse outcomes with patients concomitantly receiving both clopidogrel and a PPI. However, these findings are prone to confounding bias. In all of these registry analyses, in many important ways, patients receiving a PPI were older and sicker than those who were not receiving a PPI, and in one analysis, there was increased risk associated with the concomitant use of clopidogrel and all PPIs regardless of CYP2C19 metabolism,^10^ indirectly supporting the presence of confounding. Similarly, Blackburn and colleagues^22^ have reported that acid‐suppressive drug use with either histamine blockers or PPIs was significantly associated with hospitalization for MI or acute coronary syndrome regardless of whether patients were receiving clopidogrel, also supporting the existence of confounding in many of these observational analyses. The CREDO trial supports this hypothesis in that PPI use was associated with the 1‐year primary end point, regardless of treatment assignment to clopidogrel or placebo.

Although these data taken together primarily describe a confounding effect of PPI use, a direct cardiotoxic effect of PPIs cannot be excluded from these observational studies. Shillinger and colleagues,^23^ for example, have demonstrated that pantoprazole and omeprazole have negative effects on myocardial contractility. An analysis by Charlot and colleagues^23–24^ revealed that PPI use was associated with adverse cardiovascular events in heart failure patients irrespective of treatment with clopidogrel. Furthermore, Bell and colleagues have identified an association between PPI use and all‐cause mortality in institutionalized elderly patients, whereas Schmidt and colleagues identified that PPI use was associated with adverse cardiovascular outcomes in patients receiving PCI, despite adjustment for potential confounding variables in both analyses.^13,25^ Perhaps the greatest evidence against such a direct cardiotoxic effect, however, is the Clopidogrel and the Optimization of Gastrointestinal EvENTs (COGENT) trial, in which all patients received clopidogrel; patients randomly assigned to PPIs did not have more frequent cardiovascular events.

In contrast to CREDO, the findings in the CAPRIE trial are unexpected and represent the first subgroup analysis from a randomized trial that suggests a risk of PPI use when administered to patients assigned to clopidogrel but not the comparator antiplatelet agent whose metabolism is not believed to be affected by a PPI. These findings stand alone and are discordant with both prospective, randomized trial data as well as retrospective, post hoc analyses of existing randomized trial data. O'Donoghue and colleagues^5^ identified no significant association between PPI use and adverse outcomes in either clopidogrel‐ or prasugrel‐treated patients in the TRITON trial. Similarly, PPI use did not significantly influence outcomes in ticagrelor‐ or clopidogrel‐treated patients in the PLATO trial.^21^ Finally, the prospective, randomized trial COGENT‐1 did not identify an excess of adverse cardiovascular events with a novel drug formulation of clopidogrel and omeprazole compared with clopidogrel only.^14^

There are several potential explanations for the discordant findings observed in CAPRIE. The identification of an association between PPI use and adverse outcomes in clopidogrel‐treated patients, but not aspirin‐treated patients, is consistent with the mechanistic potential for this interaction demonstrated previously.^2,26–28^ The treatment groups in CAPRIE were clopidogrel monotherapy versus aspirin monotherapy; other data examining a PPI–clopidogrel interaction have been collected in patients receiving the combination of clopidogrel and aspirin versus a comparator. It is possible that a weak but real adverse interaction between clopidogrel and PPIs is less evident with the administration of aspirin, which was demonstrated in CAPRIE but not in the other trials because they involved the administration of dual antiplatelet therapy. It is also possible that the increase in adverse events was the play of chance in a small number of patients.

In summary, the findings from the CREDO trial support the hypothesis that PPI use is a significant confounder associated with adverse cardiovascular outcomes and that PPIs do not reduce the clinical efficacy of clopidogrel. This conclusion is reinforced by the results of our meta‐analysis. However, the analyses of CAPRIE are consistent with the hypothesis that PPIs might reduce the efficacy of clopidogrel, at least in patients receiving clopidogrel alone (without aspirin), and support the need for further study of patients receiving monotherapy with clopidogrel.
